# Drug-Eluting Balloons and Drug-Eluting Stents in Diabetic Patients Undergoing Percutaneous Coronary Intervention Due to Restenosis—DM-Dragon Registry

**DOI:** 10.3390/jcm13154464

**Published:** 2024-07-30

**Authors:** Piotr Niezgoda, Michał Kasprzak, Jacek Kubica, Łukasz Kuźma, Rafał Januszek, Sylwia Iwańczyk, Brunon Tomasiewicz, Jacek Bil, Mariusz Kowalewski, Miłosz Jaguszewski, Maciej Wybraniec, Krzysztof Reczuch, Sławomir Dobrzycki, Stanisław Bartuś, Maciej Lesiak, Mariusz Gąsior, Rafał Wolny, Adam Witkowski, Robert Gil, Bernardo Cortese, Fabrizio D’Ascenzo, Wojciech Wojakowski, Wojciech Wańha

**Affiliations:** 1 Department of Cardiology and Internal Medicine, Nicolaus Copernicus University in Toruń, L. Rydygier Collegium Medicum in Bydgoszcz, 85-094 Bydgoszcz, Poland; 2 Department of Invasive Cardiology, Medical University of Bialystok, 15-276 Bialystok, Poland; 3 Faculty of Medicine and Health Sciences, Andrzej Frycz Modrzewski Cracow University, 30-705 Kraków, Poland; 4 1st Department of Cardiology, Poznan University of Medical Sciences, 61-701 Poznan, Poland; 5 Centre for Heart Disease, Department of Heart Disease, University Hospital Wroclaw, Wroclaw Medical University, 50-556 Wroclaw, Poland; 6 Department of Cardiology, National Medical Institute of the Ministry of Interior and Administration, Wołoska 137 Str., 02-507 Warsaw, Poland; 7 Thoracic Research Centre, Collegium Medicum Nicolaus Copernicus University, Innovative Medical Forum, 85-094 Bydgoszcz, Poland; 8 Department of Cardiac Surgery and Transplantology, National Medical Institute of the Ministry of Interior and Administration, Wołoska 137 Str., 02-507 Warsaw, Poland; 9 Cardio-Thoracic Surgery Department, Heart and Vascular Centre, Maastricht University Medical Centre, Cardiovascular Research Institute Maastricht (CARIM), 6229 HX Maastricht, The Netherlands; 10 First Department of Cardiology, Medical University of Gdansk, 80-210 Gdansk, Poland; 11 First Department of Cardiology, School of Medicine in Katowice, Medical University of Silesia, 40-287 Katowice, Poland; 12 Department of Cardiology, Jagiellonian University Medical College, 30-688 Krakow, Poland; 13 Third Department of Cardiology, Medical University of Silesia, 41-800 Zabrze, Poland; 14 Department of Interventional Cardiology and Angiology, National Institute of Cardiology, 04-628 Warsaw, Poland; 15 Fondazione Ricerca e Innovazione Cardiovascolare, 20143 Milano, Italy; 16 DCB Academy, 20136 Milano, Italy; 17 Division of Cardiology, Department of Internal Medicine, Cittàdella Salute e della Scienza, University of Turin, 10126 Turin, Italy; 18 Department of Cardiology and Structural Heart Diseases, Medical University of Silesia, 40-055 Katowice, Poland

**Keywords:** DEB, DES, restenosis, diabetes mellitus, in-stent restenosis (ISR)

## Abstract

**Background**: The rate of in-stent restenosis (ISR) is decreasing; however, it is still a challenge for contemporary invasive cardiologists. Therapeutic methods, including drug-eluting balloons (DEBs), intravascular lithotripsy, excimer laser coronary atherectomy, and imaging-guided percutaneous coronary intervention (PCI) with drug-eluting stents (DES), have been implemented. Patients with diabetes mellitus (DM) are burdened with a higher risk of ISR than the general population. **Aims**: DM-Dragon is aimed at evaluating the clinical outcomes of ISR treatment with DEBs vs. DES, focusing on patients with co-existing diabetes mellitus. **Methods**: The DM-Dragon registry is a retrospective study comprising data from nine high-volume PCI centers in Poland. A total of 1117 patients, of whom 473 individuals had DM and were treated with PCI due to ISR, were included. After propensity-score matching (PSM), 198 pairs were created for further analysis. The primary outcome of the study was target lesion revascularization (TLR). **Results**: In DM patients after PSM, TLR occurred in 21 (10.61%) vs. 20 (10.1%) in non-diabetic patients, *p* = 0.8690. Rates of target vessel revascularization (TVR), target vessel myocardial infarction, device-oriented composite endpoint (DOCE), and cardiac death did not differ significantly. Among diabetic patients, the risk of all-cause mortality was significantly lower in the DEB group (2.78% vs. 11.11%, HR 3.67 (95% confidence interval, CI) [1.01–13.3), *p* = 0.0483). **Conclusions**: PCI with DEBs is almost as effective as DES implantation in DM patients treated for ISR. In DM-Dragon, the rate of all-cause death was significantly lower in patients treated with DEBs. Further large-scale, randomized clinical trials would be needed to support these findings.

## 1. Introduction

The rapid development of interventional cardiology and construction of new-generation drug-eluting stents (DES) has significantly reduced the rate of in-stent restenosis (ISR) in treated vessels. However, its rate still ranges from 5 to 10% [1]. Due to its unpredictable clinical consequences, including death, acute coronary syndromes (ACS), unscheduled revascularization or readmissions, large-scale efforts have been made to establish effective methods of both prevention and treatment of ISR [2]. Currently proposed approaches of ISR therapy comprise usage of drug eluting balloons (DEB), vascular brachytherapy, excimer laser coronary atherectomy, intravascular lithotripsy, another DES implantation all guided by intravascular imaging with intravascular ultrasound or optical coherent tomography. If none of these methods are applicable, coronary artery by-pass grafting (CABG) can be performed [3]. The rates of ISR and restenosis after balloon angioplasty have been found to be significantly higher in diabetic patients than in the general population (55% vs. 20%, *p* = 0.001) [4]. Consistently, in another study, stent-edge restenosis occurred more frequently in individuals with diabetes (20.3% vs. 9.2%, *p* = 0.019) [5]. Several potential mechanisms of ISR in diabetes mellitus (DM) patients have been proposed. Among them, the abnormal activity of vascular smooth muscle cells; more aggressive and rapidly progressing atherosclerosis; the impairment of particular glycoproteins, such as plasminogen activator inhibitor-1; increased platelet aggregation resulting from insulin-resistance; and hyperinsulinemia need to be mentioned due to their most pronounced impact on the risk of ISR [6]. The optimal treatment of ISR in the general population was evaluated in the DEB-Dragon registry, a large-scale multicenter study including 1117 participants, which showed comparable clinical outcomes, i.e., cardiac mortality, rate of recurrent myocardial infarctions (MIs), target lesion revascularization (TLR) or a device-oriented composite endpoint between patients treated with thin DES vs. drug-eluting balloons (DEBs) [7]. The DM-Dragon is a sub-analysis of the DEB-Dragon registry (ClinicalTrials.gov Identifier: NCT 04415216) aimed at evaluating the safety and efficacy of DEBs vs. thin-strut DES in diabetic patients. The DM-Dragon study focuses on the clinical outcomes of percutaneous coronary interventions (PCIs) with DEBs and DES in diabetic patients in comparison with non-DM patients. Additional analyses evaluating the safety of DEB vs. DES were conducted in study subpopulations including active smokers, history of chronic kidney disease (CKD), males vs. females, index hospitalization myocardial infarction (MI) or preselected procedural aspects such as location of the treated lesion, bifurcation angioplasty etc. were performed.

## 2. Materials and Methods

**Figure 1:**
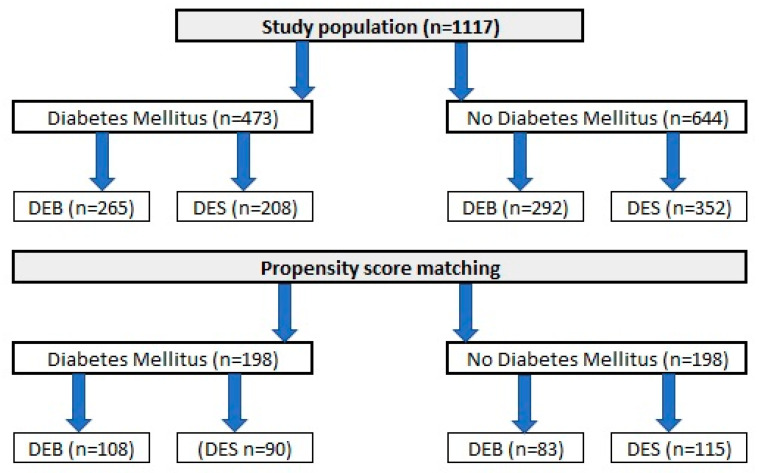
A DM-Dragon study flowchart.

A total of 1117 patients, who underwent PCI due to ISR in one of nine high-volume PCI centers in Poland, were included in the analysis. Similar to the original DEB-Dragon registry [7], patients were divided into study arms as follows: (1) patients treated with thin DES, defined as DES with a strut thickness below 100 µm (n = 560), and (2) patients treated with DEBs (n = 557). A complete list of thin DES and DEBs used in the study is presented in Appendix A. Only patients who underwent PCI in native arteries and were not treated with both methods in the same procedure were included. Moreover, patients with repeated PCI with stent implantation causing the formation of multiple stent layers were also excluded from analysis. Data on clinical outcomes throughout a 3-year follow-up period were obtained from each center’s clinical documentation, and we used the databases of the National Health Fund and follow-up phone calls if needed. The design of the study was approved by The Bioethics Committee of The Medical University of Silesia (approval number PCN/0022/KB/171/20). Because of the retrospective character of analysis, no written informed consent was required. Data of each study participant were anonymized. The study flow chart is presented in Figure 1.

## 3. Endpoints

Study endpoints in the DM-Dragon registry were defined identically to those used in the main trial, DEB-Dragon [7]; however, all outcomes were analyzed in diabetic vs. non-diabetic participants. Target lesion revascularization (TLR) was the primary endpoint of the study. A composite of cardiac death, TLR, target vessel MI (device-oriented composite endpoint—DOCE), as well as target lesion revascularization (TVR), MI and cardiac death were defined as secondary endpoints.

## 4. Statistical Analysis

The statistical analysis was carried out using the Statistica 13.0 package (TIBCO Software Inc, Palo Alto, CA, USA). Continuous data were presented as medians with inter-quartile ranges (first quartile (Q1)–third quartile (Q3). Qualitative data were presented as values and percentages. The normality of data distribution was assessed with the Shapiro–Wilk test. Since the variables were not normally distributed, comparisons of continuous variables between groups were performed with the Mann–Whitney unpaired rank sum test. Categorical variables were compared using the χ2 or Fisher exact test. Kaplan–Meier survival curves were created for time-to-event data, and data were compared with the log-rank test. Due to the inhomogeneity of the population enrolled in the study, propensity-score matching (PSM) was performed. The variables included in the PSM were age, gender, CKD, dialysis, hypertension, hyperlipidemia, chronic obstructive pulmonary disease (COPD), atrial fibrillation (AF), active smoking, previous MI, previous coronary artery bypass surgery (CABG), family history of coronary artery disease (CAD), peripheral artery disease (PAD), stable angina on admission, STEMI on admission, left ventricle ejection fraction, three-vessel disease, left main PCI, circumflex artery PCI, bifurcation lesion, thrombus, calcifications, and residual stenosis post-PCI. Due to the fact that DM-Dragon focused on the clinical outcomes of DEB vs. DES therapy in DM patients, the diagnosis of DM was not included in the PSM, contrary to the original DEB-Dragon registry [7]. After PSM, a total of 198 pairs were generated. Cox regression analysis was performed for long-term follow-up event rates. The results of the Cox regression analysis were presented as hazard ratios (HRs) and 95% confidence intervals (CIs). *p* values < 0.05 were considered significant.

## 5. Results

Taking into account the baseline characteristics of the enrolled participants (Table 1), significant differences were observed in body weight and its derivative, body mass index. Diabetic patients more commonly suffered from CKD, arterial hypertension, PAD and AF. Moreover, in diabetic patients, UA was diagnosed more often. In invasive diagnostics, the rate of a three-vessel disease was significantly higher in diabetic patients. Also, the percentage of patients with bifurcation lesions was significantly higher in cases of co-existing diabetes, and lesions were treated with longer stents.

Of the 1117 patients included in the analysis, 473 (42.3%) participants had diabetes. A 3-year follow-up (Table 2) showed no significant difference in the primary endpoint between the study arms in the unselected cohort (57 [12.05%] vs. 70 [18.87%], *p* = 0.5389) or post-PSM (20 [10.1%] vs. 21 [10.61%], *p* = 0.8690) for DM and non-DM patients, respectively. The rates of MI and all-cause death were higher among diabetic patients in the unselected cohort (70 [14.8%] vs. 52 [8.07%], *p* = 0.0004 and 43 [9.09%] vs. 28 [4.35%], *p* = 0.0013 for MI and all-cause death, respectively). However, differences in these outcomes turned out to be insignificant after PSM. The remaining outcomes, including DOCE, TVR, cardiac death and target vessel MI, did not differ significantly between the study arms neither in the unselected population nor after PSM.

**Figure 2:**
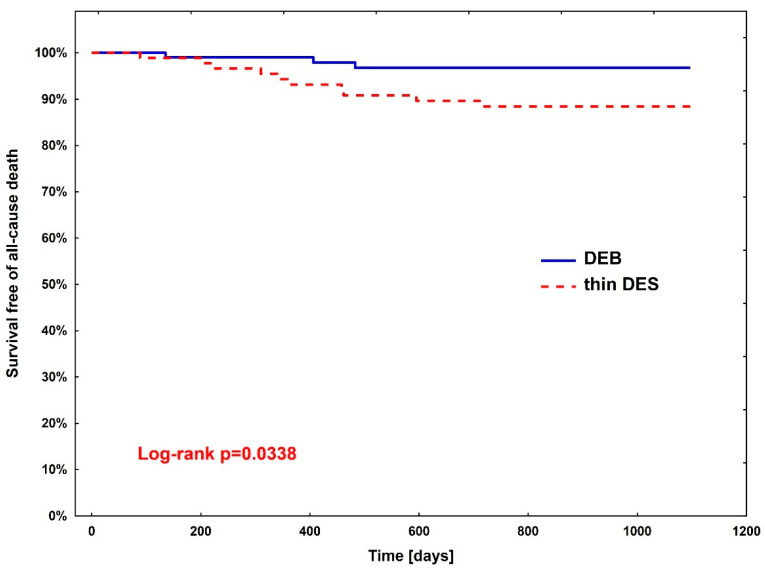
Kaplan–Meier curve for all-cause death between diabetic patients treated with DEBs (group 1) vs. DES (group 2).

Rates of clinical endpoints (Table 3) were compared between patients treated with DEBs vs. DES after PSM. A significant difference was observed only in all-cause death, where the superiority of DEBs over DES was shown (hazard ratio (HR)—3.67, 95% CI (1.01–13.3; *p* = 0.0483)). The Kaplan–Meier curve for all-cause death is presented in Figure 2.

**Figure 3:**
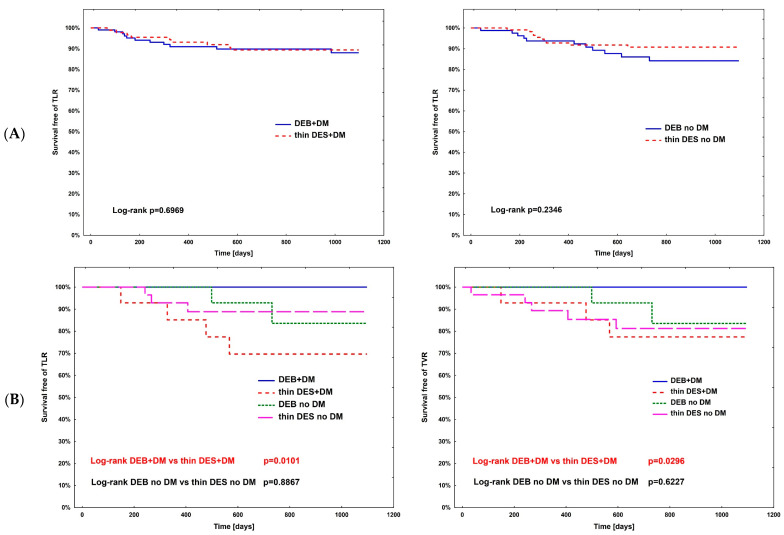

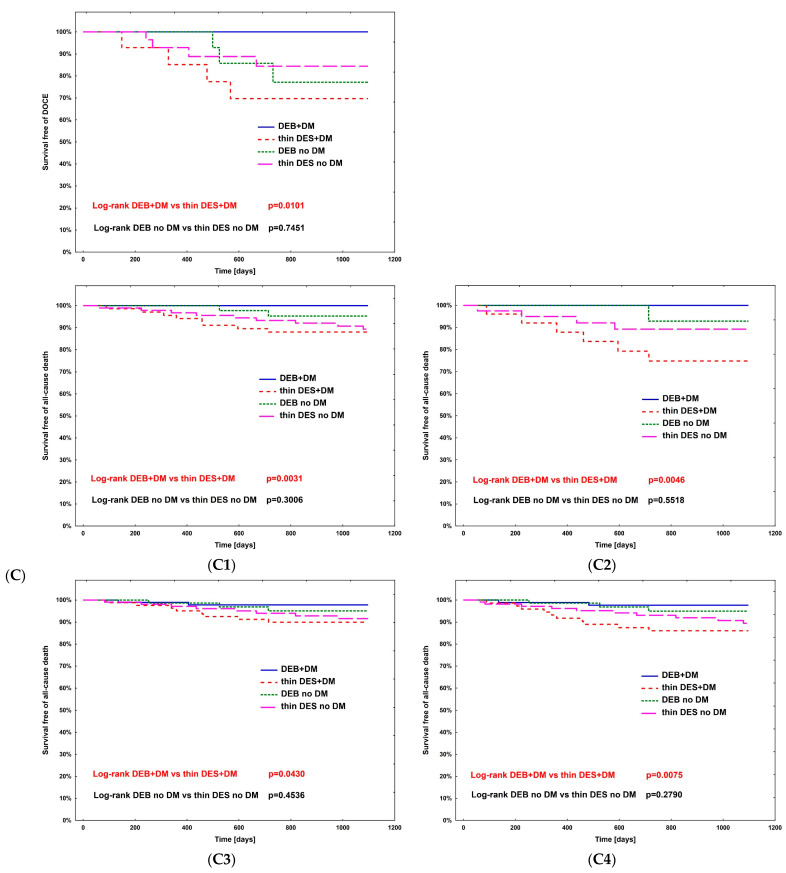

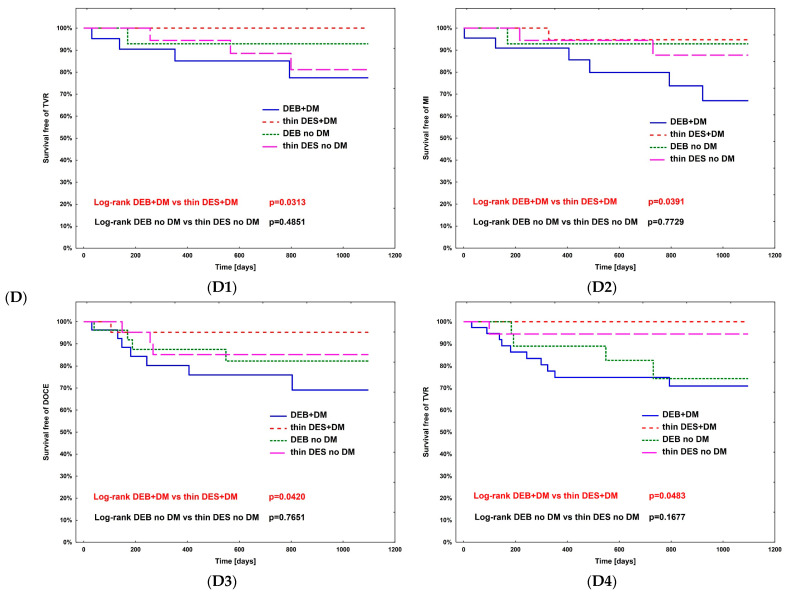
Kaplan–Meier curves for primary endpoint of DM Dragon (**A**) and preselected subpopulation analysis of DEBs vs. DES use long-term outcomes (**B**–**D**). (**B**) Active smokers. (**C**) All-cause death—superiority of DEBs over DES: (**C1**) No MI; (**C2**) Female; (**C3**) Arterial hypertension; (**C4**) Other than LM PCI. (**D**) Superiority of DES over DEBs: (**D1**) TVR—bifurcation lesions; (**D2**) MI—bifurcation lesions; (**D3**) DOCE—current MI; (**D4**) TVR—CKD patients.

The hazard ratio for TLR (Table 4) was calculated using Cox regression analysis in all subpopulations; however, no significance was observed. The complete comparison of DEBs vs. DES in diabetic and non-diabetic subgroups is presented in Table 5. After PSM, the rates of study outcomes between DES vs. DEBs in diabetic and non-diabetic subgroups including current MI vs. no MI, current ACS vs. CCS, males vs. females, CKD vs. no-CKD, hypertension vs. no hypertension, active smoking vs. non-smokers, bifurcation lesion vs. no-bifurcation, and LM PCI vs. no-LM PCI revealed no significant differences in the non-diabetic population. In DM patients, in terms of the primary endpoint of the study, the superiority of DEBs over DES was observed only in active cigarette smokers (*p* = 0.0101). Actively smoking DM patients more often experienced TVR and DOCE (*p* = 0.0296 and *p* = 0.0101, respectively). Also, more favorable outcomes were associated with the use of DEBs with respect to all-cause death in several analyzed subgroups, including females, non-smokers, and patients without bifurcation lesions, without LM PCI and who did not present with an acute MI. On the other hand, the superiority of DES over DEBs was observed in terms of TVR in CKD patients (*p* = 0.0483), as well as TVR and MI in bifurcation lesions (*p* = 0.0313 and *p* = 0.0391, respectively). Long-term observation also revealed less episodes of DOCE in patients treated for MI (*p* = 0.0420); however, as mentioned above, the superiority of DEBs over DES was significant in active smokers. Kaplan–Meier curves for the primary endpoints of the study and selected significant differences between study arms are presented in Figure 3A–D.

## 6. Discussion

As mentioned above, diabetic patients undergoing PCI are more predisposed to ISR when compared with the general population; therefore, the optimization of measures undertaken during invasive procedures is of a great importance. In the DM-Dragon study, active smokers diagnosed with DM had a significantly higher risk of TLR, TVR and DOCE if they were treated with DES implantation. On the other hand, in this study, diabetic patients undergoing the angioplasty of bifurcation lesions with DEBs were predisposed to TVR and MI when compared with DES implantation. The superiority of DES was also observed in CKD patients in terms of TVR rate and in terms of DOCE if an acute MI was currently diagnosed. Additionally, in the proposed study, rates of TLR did not differ significantly in DM vs. no DM patients treated with DEBs or DES due to ISR. In a subpopulation analysis, no significant difference was observed in terms of TLR rate in CKD vs. no CKD patients. All-cause mortality turned out to be significantly lower in DM patients treated with DEBs when compared with those who received DES.

A study by Alexandrescu et. al., which aimed to examine potential risk factors of ISR, proved the significant influence of active smoking (RR 1.63, 95% confidence interval [CI] 1.25–2.13, *p* = 0.001), arterial hypertension (RR 1.86, 95% CI 1.41–2.45, *p* = 0.001), DM (RR 1.83, 95% CI 1.42–2.36, *p* = 0.001) and CKD (RR 1.90, 95% CI 1.53–2.36, *p* = 0.001) on the rate of ISR post-PCI [8]. Also, compared to those who never smoked, individuals with a history of current or previous smoking turned out to have significantly higher risk of ISR in a study by Megaly et. al. (odds ratio [OR] 10.085, 95% CI 1.495–68.038, *p* = 0.018) [9]. In a study by Jonas et. al., patients with CKD who were treated with DEB had a higher risk of major adverse cardiac events (MACEs) (23.8% vs. 13.8%, *p* < 0.005 and MI 15.9% vs. 3.8%, *p* < 0.001) than those without CKD. Similar to the DM-Dragon study, the rate of TLR was similar in CKD vs. no-CKD patients [10]. In a large analysis of PCI outcomes (n = 3 187 404) in CKD patients (11%), there was a significant increase in in-hospital mortality, periprocedural hemorrhage, and in-hospital stay (*p* < 0.001 for each parameter). Another study, by Wang et. al., which compared outcomes of DEB vs. DES use in patients with ISR, found DM, as well as at least three stent layers and re-DES implantation, to be independent risk factors of MACEs for recurrent ISR. Contrary to DM-Dragon, the same work indicates that the risk of MACEs and TLR in recurrent ISR is significantly higher in patients treated with DES than in those who received DEBs (17.2% vs. 32.9%; *p* = 0.02 and 15.1% vs. 27.8%; *p* = 0.04, respectively) [11]. A recent review and meta-analysis by Murphy et. al. proved the utility of small-vessel angioplasty with DEBs. Patients treated with DEBs in comparison with DES had significantly lower rates of non-fatal MI at the 1-year timepoint, which was accompanied with the reduction of major bleeding episodes [12]. A DARE study by Claessen et. al. that compared clinical outcomes and angiographic parameters after PCI using paclitaxel-coated balloons (PCBs) vs. everolimus-eluting stents (EESs) showed similar major adverse events, including death, TVR and target vessel MI in diabetic patients between the study arms (MACE: 11.9% vs. 17.4% for PCBs and EESs, respectively, *p* = 0.44) [13]. Similar MACE rates at 1 year in both diabetic and non-diabetic patients treated with paclitaxel-eluting stents and PCBs were also observed in the BELLO trial by Giannini et al. (13.2% vs. 25%, *p* = 0.194 for DES and PCBs, respectively, in DM patients and 11.8% vs. 14.3%, *p* = 0.699 in no-DM ones) [14]. Another comparison of DEBs and DES in DM patients treated for ISR or de novo lesions performed by Verdoia et. al. [15] showed comparable clinical outcomes between study arms (MACE: 21.6% vs. 17.3%, HR (95% CI) 1.51 [0.46–4.93], for DEBs and DES, respectively, *p* = 0.50), but overall mortality was significantly lower in the DEB group (3.6% vs. 10.9%; HR (95% CI) 0.27 [0.08–0.91], *p* = 0.03). These results are consistent with findings from the current DM-Dragon study, where the superiority of DEBs vs. DES was observed for all-cause death in DM patients (2.78% vs. 11.11%, HR 3.67 (95% CI) [1.01–13.3), *p* = 0.0483). A meta-analysis of six clinical studies (a total of 847 diabetic patients) by Kui et. al. aimed to compare clinical outcomes of small-vessel PCI with DEBs vs. DES. Treatment with DEBs was associated with a lower risk of MACEs (RR, 0.60; 95% CI: 0.39–0.93; *p* = 0.02), MI (RR, 0.42; 95% CI, 0.19–0.94; *p* = 0.03), TLR (RR, 0.24; 95% CI, 0.08–0.69; *p* < 0.001) and TVR (RR, 0.33; 95% CI, 0.18–0.63; *p* < 0.001) [16].

## 7. Study Limitations

The DM-Dragon is a retrospective analysis of clinical data obtained from each PCI center, National Health Fund databases and follow-up phone calls. Despite a relatively large baseline population (n = 1117), PSM resulted in a noticeable reduction of participants included in the final analysis of study outcomes (198 pairs). Therefore, the numbers of some analyzed events were low, which may have influenced the obtained results. Despite the fact that the study focuses on the analysis of clinical outcomes of ISR therapy with DEBs vs. DES in DM patients, no detailed data including the duration of diabetes or its current pharmacotherapy were available.

## 8. Conclusions

In DM patients treated for ISR, PCI with DEBs has a similar effectiveness to DES implantation in terms of the rate of adverse events, including TLR, TVR, MI or cardiac death. However, patients treated with DEBs had a lower risk of all-cause death when compared with those who underwent PCI with DES implantation. The results of the DM-Dragon study may be useful to lay the foundations for the further widespread use of such a therapeutic approach in DM patients undergoing PCI due to restenosis. PCI with DEBs allows us to avoid the multiplication of stent layers in the treated vessels, which undoubtedly makes the procedure beneficial for patients. Further development of large-scale randomized clinical trials on the efficacy of DEBs vs. DES in ISR in diabetic patients would be needed to support these findings. Taking into account the DM-Dragon results, accompanied by other worldwide data on the clinical utility of DEBs in coronary procedures, conducting further trials may be considered safe for study participants, which in turn may allow us to accelerate the enrollment of patients and reduce the potential concerns of both the patient and the investigator.

## Figures and Tables

**Table 1:** Baseline characteristics of the population of DM-Dragon trial.

	Unselected Cohort			Post-Propensity Matching		
	No DM	DM	*p* Value	No DM	DM	*p* Value
	n = 644 (57.70%)	n = 473 (42.30%)		n = 198 (50.00%)	n = 198 (50.00%)	
Age	67.53 (59.00–74.00)	66 (61.00–73.20)	0.6898	68 (61–74)	66.00 (60.00–73.52)	0.2529
Weight [kg]	80 (71–89)	84 (75–92)	0.0001	81 (71–88)	85 (75–92)	0.0045
Height [cm]	170 (165–175)	169 (164–174)	0.0593	170 (164–175)	169 (164–174)	0.5389
BMI	27.58 (25.26–30.35)	29.03 (26.51–32.35)	<0.0001	27.77 (25.95–30.12)	29.05 (26.95–32.05)	0.0019
Male	461 (71.58)	324 (68.5)	0.2650	139 (70.2)	138 (69.70)	0.9127
Ejection fraction [%]	50.00 (45.00–55.00)	50.00 (40.00–55.00)	<0.0001	50.00 (45.00–55.00)	50.00 (45.00–55.00)	0.8089
						
DEB used	292 (45.34)	265 (56.03)	0.0004	83 (41.92)	108 (54.55)	0.0120
DES used	352 (54.66)	208 (43.97)		115 (58.08)	90 (45.45)	
						
CKD	119 (18.48)	136 (28.75)	<0.0001	37 (18.69)	50 (25.25)	0.1146
Hypertension	555 (86.18)	439 (92.81)	0.0005	183 (92.42)	183 (92.42)	1.0000
Hyperlipidemia	553 (85.87)	404 (85.41)	0.8293	170 (85.86)	164 (82.83)	0.4067
COPD	47 (7.3)	47 (9.94)	0.1165	14 (7.07)	16 (8.08)	0.7041
Active smoker	139 (21.58)	84 (17.76)	0.1141	46 (23.23)	36 (18.18)	0.2149
Previous MI	389 (60.4)	310 (65.54)	0.0797	120 (60.61)	117 (59.09)	0.7584
Previous CABG	110 (17.08)	100 (21.14)	0.0861	40 (20.2)	39 (19.7)	0.9000
Family history of CAD	213 (33.13)	150 (31.85)	0.6528	77 (38.89)	69 (34.85)	0.4047
PAD	89 (13.82)	98 (20.72)	0.0023	26 (13.13)	28 (14.14)	0.7696
Atrial fibrillation	83 (12.89)	81 (17.12)	0.0481	26 (13.13)	29 (14.65)	0.6629
						
Chronic coronary syndrome	267 (41.46)	172 (36.36)	0.0850	84 (42.42)	75 (37.88)	0.3562
Unstable angina	206 (31.99)	179 (37.84)	0.0419	66 (33.33)	75 (37.88)	0.3449
STEMI	19 (2.95)	12 (2.54)	0.6778	9 (4.55)	8 (4.04)	0.8042
NSTEMI	152 (23.6)	110 (23.26)	0.8925	39 (19.7)	40 (20.2)	0.9000
1-vessel disease	388 (60.25)	263 (55.6)	0.1197	126 (63.64)	118 (59.6)	0.4084
2-vessel disease	185 (28.73)	137 (28.96)	0.9310	52 (26.26)	47 (23.74)	0.5617
3-vessel disease	69 (10.71)	71 (15.01)	0.0321	20 (10.1)	32 (16.16)	0.0742
						
Stenosis	82.5 (80–90)	80.0 (75–90)	0.9902	82.5 (80–90)	80 (75–90)	0.3886
Bifurcation lesion	100 (15.53)	100 (21.14)	0.0156	33 (16.67)	43 (21.72)	0.2019
Original stent diameter [mm]	3.00 (3.00–3.5)	3.00 (3.00–3.5)	0.4730	3.00 (3.00–3.50)	3.00 (3.00–3.50)	0.5802
Original stent length [mm]	20.00 (16.00–26.00)	22.00 (18.00–28.00)	0.0368	20.00 (17.00–28.00)	22.00 (16.50–27.50)	0.9828
LM PCI	51 (7.92)	50 (10.57)	0.1797	16 (8.08)	26 (13.13)	0.1027
LAD PCI	271 (42.08)	184 (38.90)	0.2852	80 (40.40)	74 (37.37)	0.5363
Cx PCI	134 (20.81)	114 (24.10)	0.1906	35 (17.68)	44 (22.22)	0.2577
RCA PCI	213 (33.07)	150 (31.71)	0.6311	72 (36.36)	64 (32.32)	0.3972
Residual stenosis post-PCI	38 (5.9)	34 (7.19)	0.3866	12 (6.06)	12 (6.06)	1.0000
TIMI 3 post-PCI	629 (97.67)	461 (97.46)	0.8232	194 (97.98)	194 (97,98)	0.7210
						
ISR focal	311 (48.29)	234 (49.47)	0.6968	97 (48.99)	96 (48.48)	0.9199
ISR diffuse	220 (34.16)	161 (34.04)	0.9657	64 (32.32)	66 (33.33)	0.8305
ISR proliferative	100 (15.53)	69 (15.59)	0.6648	32 (16.16)	32 (16.16)	1.0000
ISR occlusive	13 (2.02)	9 (1.9)	0.8905	5 (2.53)	4 (2.02)	1.0000

Abbreviations used: BMI—body mass index, DEB—drug-eluting balloon, DES—drug-eluting stent, CKD—chronic kidney disease, COPD—chronic obstructive pulmonary disease, MI—myocardial infarction, CABG—coronary artery bypass grafting, CAD—coronary artery disease, PAD—peripheral artery disease, STEMI—ST-elevation myocardial infarction, NSTEMI—non-ST-elevation myocardial infarction, LM—left main trunk, LAD—left anterior descending artery, Cx—circumflex artery, RCA—right coronary artery, PCI—percutaneous coronary intervention, TIMI—thrombolysis in myocardial infarction, ISR—in-stent restenosis.

**Table 2:** Long-term clinical outcomes in DM-Dragon trial.

	Unselected Cohort			Post-Propensity Matching		
	No DM	DM	*p* Value	No DM	DM	*p* Value
	n = 644 (57.7%)	n = 473 (42.3%)		n = 198 (50%)	n = 198 (50%)	
TLR	70 (10.87)	57 (12.05)	0.5389	21 (10.61)	20 (10.1)	0.8690
DOCE	83 (12.89)	77 (16.28)	0.1100	26 (13.13)	25 (12.63)	0.8808
TVR	88 (13.66)	71 (15.01)	0.5247	28 (14.14)	26 (13.13)	0.7696
Cardiac death	13 (2.02)	18 (3.81)	0.0724	5 (2.53)	5 (2.53)	1.0000
MI	52 (8.07)	70 (14.8)	0.0004	16 (8.08)	23 (11.63)	0.2378
TV MI	20 (3.11)	23 (4.86)	0.1315	6 (3.03)	8 (4.04)	0.5863
All-cause death	28 (4.35)	43 (9.09)	0.0013	13 (6.57)	13 (6.57)	1.0000

Abbreviations used: TLR—target lesion revascularization, DOCE—device-oriented composite endpoint, TVR—target vessel revascularization, MI—myocardial infarction, TV—target vessel.

**Table 3:** Summary of clinical endpoints in the DM-Dragon study.

	No Diabetes Mellitus						
	DEB (n = 83)		DES (n = 115)		HR	95% CI	*p* Value (Cox)
	n	%	n	%			
TLR	11	13.25%	10	8.70%	0.60	0.25–1.40	0.2356
TVR	13	15.66%	15	13.04%	0.77	0.36–1.61	0.4821
MI	7	8.43%	9	7.83%	0.86	0.32–2.00	0.7601
TV MI	3	3.61%	3	2.61%	0.69	0.14–3.40	0.6455
Cardiac death	1	1.20%	4	3.48%	2.57	0.29–23.06	0.3982
DOCE	13	15.66%	13	11.30%	0.65	0.30–1.40	0.2718
All-cause death	3	3.61%	10	8.70%	2.06	0.57–7.51	0.2726
	**Diabetes Mellitus**						
	**DEB (n = 108)**		**DES (n = 90)**		**HR**	**95% CI**	***p* Value (Cox)**
	**n**	**%**	**n**	**%**			
TLR	11	10.19%	9	10.00%	0.89	0.37–2.15	0.7982
TVR	15	13.89%	11	12.22%	0.78	0.36–1.71	0.5390
MI	14	12.96%	9	10.00%	0.70	0.30–1.61	0.3968
TV MI	5	4.63%	3	3.33%	0.62	0.15–2.61	0.4795
Cardiac death	1	0.93%	4	4.44%	4.24	0.47–37.97	0.1963
DOCE	13	12.04%	12	13.33%	0.98	0.45–2.15	0.9578
All-cause death	3	2.78%	10	11.11%	3.67	1.01–13.30	0.0483

Abbreviations used: TLR—target lesion revascularization, DOCE—device-oriented composite endpoint, TVR—target vessel revascularization, MI—myocardial infarction, TV—target vessel, HR—hazard ratio, CI—confidence interval.

**Table 4:** Hazard ratio for target lesion revascularization between patients treated with DEBs vs. DES in study sub-populations.

	No Diabetes Mellitus						
Target Lesion Revascularization	DEB (n = 83)		DES (n = 115)		HR	95% CI	*p* Value (Cox)
	n	%	n	%			
NO MI (n = 150)	8/57	14.04%	7/93	7.53%	0.50	0.18–1.37	0.1769
MI (n = 48)	3/26	11.54%	3/22	13.64%	1.07	0.21–5.28	0.9382
NO ACS (n = 84)	2/28	7.14%	1/56	1.79%	0.23	0.02–2.56	0.2334
ACS (n = 114)	9/55	16.36%	9/59	15.25%	0.89	0.35–2.24	0.8006
FEMALE (n = 59)	4/19	21.05%	4/40	10.00%	0.45	0.11–1.79	0.2565
MALE (n = 139)	7/64	10.94%	6/75	8.00%	0.64	0.22–1.91	0.4234
NO CKD (n = 161)	8/64	12.50%	10/97	10.31%	0.76	0.30–1.93	0.5658
CKD (n = 37)	3/19	15.79%	0/18	0.00%	n/a	n/a	n/a
NO AH (n = 15)	1/5	20.00%	0/10	0.00%	n/a	n/a	n/a
AH (n = 183)	10/78	12.82%	10/105	9.52%	0.67	0.28–1.60	0.3660
NO Active smoker (n = 152)	9/66	13.64%	7/86	8.14%	0.54	0.20–1.44	0.2180
Active smoker (n = 46)	2/17	11.76%	3/29	10.34%	0.88	0.15–5.26	0.8871
NO Bifurcation (n = 165)	10/68	14.71%	9/97	9.28%	0.58	0.23–1.42	0.2299
Bifurcation (n = 33)	1/15	6.67%	1/18	5.56%	0.75	0.05–11.99	0.8385
NO LM (n = 182)	11/76	14.47%	9/106	8.49%	0.53	0.22–1.27	0.1537
LM (n = 16)	0/7	0.00%	1/9	11.11%	n/a	n/a	n/a
	**Diabetes Mellitus**						
**Target Lesion Revascularization**	**DEB (n = 108)**		**DES (n = 90)**		**HR**	**95% CI**	***p* Value** **(Cox)**
	**N**	**%**	**N**	**%**			
NO MI (n = 150)	6/81	7.41%	7/69	10.14%	1.27	0.43–3.79	0.6661
MI (n = 48)	5/27	18.52%	2/21	9.52%	0.48	0.09–2.46	0.3776
NO ACS (n = 75)	3/32	9.38%	6/43	13.95%	1.39	0.35–5.55	0.6430
ACS (n = 123)	8/76	10.53%	3/47	6.38%	0.56	0.15–2.13	0.3983
FEMALE (n = 60)	3/35	8.57%	0/25	0.00%	n/a	n/a	n/a
MALE (n = 138)	8/73	10.96%	9/65	13.85%	1.13	0.43–2.92	0.8084
NO CKD (n = 148)	4/70	5.71%	9/78	11.54%	1.87	0.58–6.08	0.2979
CKD (n = 50)	7/38	18.42%	0/12	0.00%	n/a	n/a	n/a
NO AH (n = 15)	0/8	0.00%	2/7	28.57%	n/a	n/a	n/a
AH (n = 183)	11/100	11.00%	7/83	8.43%	0.68	0.26–1.75	0.4244
NO Active smoker (n = 162)	11/86	12.79%	5/76	6.58%	0.46	0.16–1.32	0.1469
Active smoker (n = 36)	0/22	0.00%	4/14	28.57%	n/a	n/a	n/a
NO Bifurcation (n = 155)	9/86	10.47%	8/69	11.59%	1.03	0.40–2.66	0.9564
Bifurcation (n = 43)	2/22	9.09%	1/21	4.76%	0.47	0.04–5.23	0.5421
NO LM (n = 172)	9/97	9.28%	8/75	10.67%	1.08	0.42–2.80	0.8752
LM (n = 26)	2/11	18.18%	1/15	6.67%	0.30	0.03–3.38	0.3327

Abbreviations used: ACS—acute coronary syndrome, AH—arterial hypertension, CKD—chronic kidney disease, LM—left main trunk, MI—myocardial infarction, PCI—percutaneous coronary intervention. n/a—too few events to calculate *p* value.

**Table 5:** Clinical outcomes analysis in pre-selected subgroups of patients before and after PSM. (*)—indicates superiority of DEBs, (+)—indicates superiority of DES.

	Unselected Cohort			Post-Propensity Matching	
	No DM	DM		No DM	DM
	DEB vs. DES *p* Value	DEB vs. DES *p* Value		DEB vs. DES *p* Value	DEB vs. DES *p* Value
**Target lesion revascularization**					
MI	0.9379	0.3648	no MI	0.1725	0.6648
ACS	0.8010	0.3878	CCS	0.1994	0.6432
Male	0.4231	0.8079	Female	0.2483	0.1316
CKD	0.0611	0.1149	no CKD	0.5680	0.2915
AH	0.3673	0.4202	no AH	n/a	0.1025
Active smoker	0.8867	0.0101 (*)	non-smoker	0.2146	0.1364
Bifurcation	0.8388	0.5324	no bifurcation	0.2282	0.9563
LM PCI	n/a	0.3149	no LM PCI	0.1510	0.8752
**Target vessel revascularization**					
MI	0.8813	0.1202	no MI	0.4727	0.6826
ACS	0.9983	0.0946	CCS	0.6178	0.3398
Male	0.9054	0.6313	Female	0.2483	0.4610
CKD	0.1677	0.0483 (+)	no CKD	0.8965	0.2618
AH	0.6703	0.1740	no AH	n/a	0.0377 (*)
Active smoker	0.6227	0.0296 (*)	non-smoker	0.2880	0.1332
Bifurcation	0.4851	0.0313 (+)	no bifurcation	0.2998	0.7366
LM PCI	n/a	0.0920	no LM PCI	0.3627	0.9669
**Myocardial Infarction**					
MI	0.4915	0.2488	no MI	0.1141	0.6802
ACS	0.8549	0.4581	CCS	0.3810	0.5499
Male	0.9154	0.2542	Female	0.6628	0.8575
CKD	0.9934	0.0980	no CKD	0.7180	0.9069
AH	0.8350	0.4836	no AH	0.6137	n/a
Active smoker	0.6567	0.9988	non-smoker	0.5242	0.3495
Bifurcation	0.7729	0.0391 (+)	no bifurcation	0.6248	0.7719
LM PCI	n/a	0.0906	no LM PCI	0.5768	0.7815
**Target vessel myocardial infarction**					
MI	0.7263	0.5063	no MI	n/a	0.9643
ACS	0.8853	0.7290	CCS	n/a	0.7541
Male	0.6853	0.5997	Female	0.1905	0.7373
CKD	0.1493	0.2361	no CKD	0.5577	0.4679
AH	0.9583	0.4979	no AH	n/a	n/a
Active smoker	0.3006	n/a	non-smoker	0.1850	0.4662
Bifurcation	n/a	n/a	no bifurcation	0.9898	0.8083
LM PCI	n/a	n/a	no LM PCI	0.6352	0.6063
**Cardiac death**					
MI	n/a	0.9035	no MI	0.4747	0.0769
ACS	n/a	n/a	CCS	0.6223	0.1665
Male	0.7716	0.5540	Female	0.2339	0.1111
CKD	n/a	n/a	no CKD	0.6026	0.0771
AH	0.5862	0.3048	no AH	n/a	n/a
Active smoker	n/a	n/a	non-smoker	0.1453	0.1806
Bifurcation	0.7533	0.6281	no bifurcation	0.2666	0.1405
LM PCI	n/a	n/a	no LM PCI	0.3934	0.0298 (*)
**Device-oriented composite endpoint**					
MI	0.7651	0.0420 (+)	no MI	0.2931	0.1698
ACS	0.4769	0.0818	CCS	0.6302	0.1853
Male	0.2743	0.9318	Female	0.5324	0.8649
CKD	0.1384	0.0556	no CKD	0.6271	0.1058
AH	0.3076	0.3045	no AH	0.6420	0.0176 (*)
Active smoker	0.7451	0.0101 (*)	non-smoker	0.2958	0.2433
Bifurcation	0.9361	0.5484	no bifurcation	0.2165	0.7819
LM PCI	n/a	0.1218	no LM PCI	0.1855	0.5419
**All-cause death**					
MI	0.9390	0.7847	no MI	0.3006	0.0031 (*)
ACS	0.1584	0.1799	CCS	0.9312	0.0716
Male	0.3878	0.6745	Female	0.5518	0.0046 (*)
CKD	0.2231	0.4137	no CKD	0.5310	0.0703
AH	0.4536	0.0430 (*)	no AH	0.3378	0.4034
Active smoker	0.5224	n/a	non-smoker	0.0932	0.0177 (*)
Bifurcation	0.7533	0.9116	no bifurcation	0.2730	0.0104 (*)
LM PCI	n/a	n/a	no LM PCI	0.2790	0.0075 (*)

Abbreviations used: ACS—acute coronary syndrome, AH—arterial hypertension, CCS—chronic coronary syndrome, CKD—chronic kidney disease, LM—left main trunk, MI—myocardial infarction, PCI—percutaneous coronary intervention, PSM—propensity-score matching, n/a—too few events to calculate *p* value.

## Data Availability

The original contributions presented in the study are included in the article, further inquiries can be directed to the corresponding author.

## Abbreviations

ACS	acute coronary syndrome
AF	atrial fibrillation
AH	arterial hypertension
CABG	coronary artery bypass grafting
CAD	coronary artery disease
CCS	chronic coronary syndrome
CI	confidence interval
CKD	chronic kidney disease
DEB	drug-eluting balloon
DES	drug-eluting stent
DM	diabetes mellitus
DOCE	device-oriented composite endpoint
HR	hazard ratio
ISR	in-stent restenosis
LM	left main trunk
MACE	major adverse cardiac event
MI	myocardial infarction
PAD	peripheral artery disease
PCI	percutaneous coronary intervention
PSM	Propensity-score matching
RR	risk ratio
TLR	target lesion revascularization
TVR	target vessel revascularization
UA	unstable angina

## Appendix A. The Complete List of Thin-Strut DES and Paclitaxel-Coated DEB Types Used in the Study

**Thin-Strut DES**		**Paclitaxel—DEB**	
**Name**	**Manufacturer**	**Name**	**Manufacturer**
Xience	Abbott Vascular Devices, Santa Clara, CA, USA	Agent	Boston Scientific, Natick, MA, USA
Resolute	(Medtronic CardioVascular, Santa Rosa, CA, USA	Elutax	Aachen Resonance GmbH, Aachen, Germany
Promus	Boston Scientific, Natick, MA, USA	Essentia	iVascular, Barcelona, Spain
Ultimaster	Terumo Corporation, Tokyo, Japan	In.Pact	Medtronic Vascular, Santa Clara, CA, USA
Synergy	Boston Scientific, Natick, MA, USA	Pantera Lux	Biotronik AG, Buulach, Switzerland
Orsiro	Biotronik AG, Bulach, Switzerland	Restore DEB	Cardionovum GmbH, Bonn, Germany
Alex	Balton, Warsaw, Poland	SeQuentPleaseNeo	B.Braun Interventional Group, Ltd., Melsulgen, Germany
